# Comparison of phenylephrine and norepinephrine in the management of dopamine-resistant septic shock

**DOI:** 10.4103/0972-5229.63033

**Published:** 2010

**Authors:** Gaurav Jain, D. K. Singh

**Affiliations:** **From:** Department of Anesthesia, Banaras Hindi University, Varanasi, U.P., India

**Keywords:** Dopamine, norepinephrine, phenylephrine

## Abstract

**Introduction:**

This study aims to compare two vasoconstrictors: – norepinephrine and phenylephrine – in the management of dopamine–resistant septic shock.

**Materials and Methods:**

We performed a randomized, prospective, controlled trial in 54 septic shock patients, with persistent hypotension despite adequate volume resuscitation and continued dopamine infusion ~25μg/kg/h. Patients were randomly allocated into two groups to receive either norepinephrine or phenylephrine infusion (n = 27 each) titrated to achieve a target of SBP > 90mm Hg, MAP > 75 mm Hg, SVRI > 1100 dynes.s/cm5m2, CI > 2.8 L/min/m2, DO2I > 550 ml/min/m2, and VO2I > 150 ml/min/m2 for continuous 6 h. All the parameters were recorded every 30 min and increment in dose of studied drug was done in the specified dose range if targets were not achieved. Data from pulmonary arterial and hepatic vein catheterization, thermodilution catheter, blood gas analysis, blood lactate levels, invasive blood pressure, and oxygen transport variables were compared with baseline values after achieving the targets of therapy. Differences within and between groups were analyzed using a one-way analysis of variance test and Fischer's exact test.

**Results:**

No difference was observed in any of the investigated parameters except for statistically significant reduction of heart rate (HR) (*P*<0.001) and increase in stroke volume index (SVI) (*P*<0.001) in phenylephrine group as compared to nonsignificant change in norepinephrine group.

**Conclusions:**

Phenylephrine infusion is comparable to norepinephrine in reversing hemodynamic and metabolic abnormalities of sepsis patients, with an additional benefit of decrease in HR and improvement in SVI.

## Introduction

Septic shock is one of the major causes of mortality or morbidity in intensive care units. Most deaths are associated with arterial hypotension and/or organ failure refractory to antibiotic therapy, fluid expansion, and vasopressor treatment. It may result in abnormal blood flow distribution, tissue hypoxia, and decrease in oxygen consumption.[1] Recently, attention has been focused on optimizing oxygen transport variables, specifically oxygen delivery and consumption, in the management of sepsis.[2] However, few data exist concerning the effects of specific vasopressor on oxygen transport indicators, particularly serum lactate levels.

The only previous study comparing phenylephrine and norepinephrine in septic shock patients suggest that early administration does not worsen serum lactate levels with both.[3] Literature, however, recommends the use of other pressor agents only in “dopamine-resistant” septic shock patient.[4,5] Similar comparison has not been done previously on dopamine–resistant sepsis patients to the best of our knowledge. Hence this study aims to compare two vasoconstrictors: – norepinephrine and phenylephrine – in the management of dopamine–resistant septic shock.

## Materials and Methods

After ethics committee approval, a written informed consent was obtained from 54 subjects (relatives as the subjects were in altered consciousness), admitted to the intensive care unit, presenting with septic shock unrelated to the primary focus. Inclusion criteria were persistent hypotension, evidence of one or more end organ dysfunction, infection along with two or more of the following criteria: (1) body temperature higher than 38°C or less than 36°C, (2) heart rate (HR) greater than 90/min, (3) respiratory rate greater than 20/min, or arterial CO_2_<32 mm Hg, (4) WBC count > 12000/ mm^3^, or < 4000/ mm^3^ or > 10% immature band form. Persistent hypotension was defined as:- systolic arterial blood pressure (SBP) <90 mm Hg or mean arterial pressure (MAP) < 60 mm Hg and central venous pressure (CVP) >12 mm Hg or pulmonary artery occlusion pressure (PAOP) >18 mm Hg, despite adequate fluid resuscitation and continuous infusion of pharmacological doses of dopamine ~25 μg/kg/min for 1 h.

Exclusion criteria were acute coronary artery disease or underlying cardiac dysfunction [cardiac index (CI) <2.2 l/min/m^2^], acute mesenteric ischemia, severe liver disease (Child Pugh grade C), chronic renal failure, and uncorrected shock due to blood loss. The identification of septic shock was made according to suggestion of Society of Critical Care Medicine (SCCM)/European Society of Intensive Care Medicine (ESICM)/American College of Chest Physicians (ACCP) –/– American Thoracic Society (ATS)/– Surgical Infection Society (SIS) International Sepsis Definitions Conference.

The demographic details, cause of sepsis, and APACHE II scoring were recorded. All subjects were mechanically ventilated with the target to maintain PaO_2_ more than 60 mm Hg and PaCO_2_ in a range of 35 – 40 mm Hg. Sedation and analgesia was given by fentanyl and midazolam.

Pulmonary artery and hepatic vein catheterization was performed using 7 F pulmonary artery catheter (Edwards Life sciences, USA). Any complications during catheter insertion were recorded. Parameters measured included PAOP, CI, stroke volume index (SVI), systemic vascular resistance index (SVRI), and hepatic vein oxygen saturation (HVOS). Hemodynamic monitoring was done using continuous electrocardiogram (ECG) and invasive arterial pressure (Becton Dickinsin DTX plus DT- 6012 transducer, USA). The MAP and PAOP were measured at end expiration. The CI was measured using the continuous thermodilution technique (Vigilance^®^ II; Edwards Life sciences, USA). Blood gas analysis was performed with automated blood gas analyzer (Cobas B 121, Roche diagnostics GmbH, Germany). Blood lactate concentrations were determined using an enzymatic method (YSI 2300 Stat Plus, Yellow Springs, USA). Oxygen transport indices [delivery index (DO2I) and consumption index (VO2I)] were calculated based on the Fick equation. Maximal infusion requirement of studied drug, no of responders, survivors, and urine output (UO) were also recorded.

Patients enrolled in the study were randomly allocated to two groups, using computer generated random numbers according to studied drug used [Table 1]. Baseline parameters were recorded at the moment when the infusion of studied drug was initiated. This was taken as baseline 0 hour (study entry) reading. The operator who manipulated the syringe pump knew about the group allocation and what the set aliquot for that drug were. The assessment of outcome was done by another physician blinded to the studied drug as per protocol of our study.

**Table 1:** Study design

	Group-I (27 patients)	Group-II (27 patients)
Drug used	Norepinephrine	Phenylephrine
Dose range	0.5–3.5 μg/kg/min	0.5–8.5 μg/kg/min
Increments	0.5 μg/kg/min	1 μg/kg/min
Time interval	30 min	30 min

The target of therapy was to achieve all the following parameters:

- SBP > 90 mm Hg
- MAP > 75 mm Hg
- SVRI > 1100 dynes.s/cm^5^m^2^
- CI > 2.8 L/min/m^2^
- DO_2_I > 550 ml/min/m^2^
- VO_2_I > 150 ml/min/m^2^

All the parameters were recorded every 30 min and increment in dose of studied drug was done if targets were not achieved. To maintain a CVP in the range of 8–15 mm Hg and the PAOP between 12–18 mm Hg, serial IV fluid challenge were given throughout the study duration.[1] Dopamine infusion was continued at a rate of 25 μg/kg/min throughout the study duration. The “responder” to the studied drug was defined as the subject who achieved and maintained all the predefined targets of therapy for a period of continuous 6 h, in the specified dose range [Table 1]. The post-treatment parameters were recorded at termination of study, on achievement of target of therapy for continuous 6 h in responders, or at maximum dose of studied drug in non-responders.

To detect a 20% difference in the measured variables with an expected standard deviation of 25% estimated from initial pilot observations, with 80% power (20% beta error) and 95% confidence level (5% alpha error), a sample size of 25 subjects per group was required. The admission rate of septic shock patients in our ICU is about 8–10 per month. The duration of the study was 12 months that is from August 2008 to July 2009. All septic shock patients admitted in the ICU during the above period were screened for our study, with the target to include at least 25 cases in each group.[6] Sample size was calculated using power and sample size calculator by the Department of Biostatics, Vanderbilt University, USA.

Statistical analysis was done on SPSS 13.0 statistic software. Comparison of APACHE II scoring and sex distribution was done by using Fischer's exact test. Other parameters were compared by one-way analysis of variance test. For the entire test results, α-error probability of *P* < 0.05 was considered statistically significant.

## Results

Out of 98 subjects screened for the study in the above duration, 60 subjects meeting the inclusion and exclusion criteria were initially randomized into two study groups. Three subjects in each group were later excluded from the study, due of protocol violation. Thus, 54 subjects completed the study successfully.

The demographic details of the subjects (age, weight, sex), cause of septic shock, and APACHE II score (18 ± 3.34 in group 1 as compared to 19.04 ± 3.73 in group 2) were found to be non significant between the studied groups [Table 2].

**Table 2:** Comparison of demographic profile, APACHE II, and cause of shock among study groups

Demographic variables	Group	Mean	Standard Deviation	*P*
Age (yrs)	1	42.88	5.39	0.178
	2	45.29	7.41	
Weight (kg)	1	64.55	7.06	0.486
	2	63.22	6.90	
APACHEII	1	17.66	3.43	0.111
	2	19.11	3.11	
	Group 1		Group 2	*P*
Sex distribution (males)	15/27 (55.56%)		13/27 (48.15%)	0.39
ARDS	8/27 (29.63%)		7/27 (25.93%)	0.5
Pneumonia	4/27 (14.81%)		5/27 (18.52%)	0.5
Abscess	5/27 (18.52%)		7/27 (25.93%)	0.32
Poly trauma	7/27 (25.93%)		6/27 (22.22%)	0.5
Necrotizing fasciitis	3/27 (11.11%)		2/27 (7.41%)	0.82

There was no complication during pulmonary artery/hepatic vein catheterization. There was no significant difference in the baseline variables between studied groups [Table 3].

**Table 3:** Pretreatment parameters

Parameters	Group	Mean	Standard Deviation	*P*
DO2I	2	708.85	25.46	0.20
(ml/min/m^2^)	1	700.33	20.77	
HVOS	2	60.66	1.73	0.12
(%)	1	59.81	2.58	
LACTATE	2	3.44	0.64	0.83
(mmol/l)	1	3.40	0.74	
CI	2	5.02	0.56	0.58
l/min/m^2^	1	4.97	0.30	
SVRI	2	676.59	12.37	0.26
(dyne.s/cm^5^m^2^)	1	680.37	13.20	
PAOP	2	15.07	1.03	0.41
(mm Hg)	1	15.40	1.52	
SBP	2	74.59	5.17	0.46
(mm Hg)	1	73.66	4.69	
HR	1	151.74	7.62	0.68
(beats/min)	2	152.66	7.28	
UO	1	0.17	0.07	0.89
(ml/kg/h)	2	0.17	0.07	
VO2I	2	166.25	10.4	0.22
(ml/min/m^2^)	1	170.03	7.14	
MAP	2	48.96	3.36	0.21
(mm Hg)	1	47.55	4.30	
SVI	2	43.54	1.43	0.32
(ml/m^2^)	1	44.04	1.61	

There was no considerable difference in amount of fluid infusion given during the study phase in both groups [Table 4].

**Table 4:** Comparison between pre and post treatment parameters

Group	Parameters	Baseline(A)/Post(B) Treatment	Mean	Standard Deviation	*P*
1	DO2I	A	700.33	20.77	<0.001
	(ml/min/m^2^)	B	800.85	13.66	
2		A	708.85	25.46	<0.001
		B	795.29	11.85	
1	VO2I	A	170.03	7.14	<0.001
	(ml/min/m^2^)	B	200.00	8.00	
2		A	166.25	10.40	<0.001
		B	195.70	6.84	
1	SVRI	A	680.37	13.20	<0.001
	(dyne.s/cm5m^2^)	B	1260.48	69.30	
2		A	676.59	12.37	<0.001
		B	1226.81	83.19	
1	PAOP	A	15.40	1.52	<0.05
	(mm Hg)	B	17.66	1.90	
2		A	15.07	1.03	0.34
		B	15.29	0.91	
1	HR	A	151.74	7.62	<0.001
	(beats/min)	B	115.66	7.46	
2		A	152.66	7.28	0.44
		B	150.48	12.72	
1	LACT	A	3.40	0.74	<.05
	(mmol/l)	B	2.90	0.35	
2		A	3.44	0.64	0.001
		B	2.87	0.39	
1	SBP	A	73.66	4.69	<0.001
	(mm Hg)	B	104.22	13.54	
2		A	74.59	5.17	<0.001
		B	111.66	11.20	
1	SVI	A	44.04	1.61	<0.001
	(ml/m^2^)	B	54.68	1.28	
2		A	43.54	1.43	0.167
		B	44.18	1.29	
1	CI	A	4.97	0.30	0.144
	(l/min/m^2^)	B	5.08	0.31	
2		A	5.02	0.56	0.51
		B	5.09	0.19	
1	MAP	A	47.55	4.30	<0.001
	(mm Hg)	B	76.14	7.46	
2		A	48.96	3.36	<0.001
		B	77.85	6.67	
1	HVOS	A	59.81	1.96	<0.001
	(%)	B	66.88	1.76	
2		A	60.66	1.73	<0.001
		B	67.4	2.06	
1	UO	A	0.17	0.07	<0.001
	(ml/kg/h)	B	0.48	0.07	
2		A	0.17	0.07	<0.001
		B	0.51	0.07	
1	IV Fluid infusion		3.41	0.18	0.283
2	(liter)		3.5000	0.34418	

**Figure 1:**
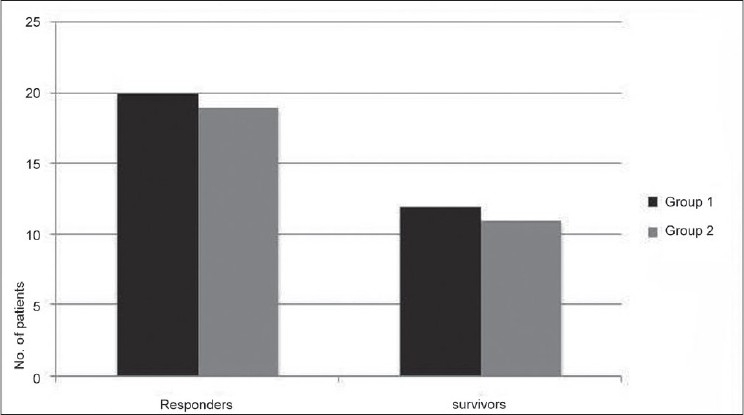
Patient Outcome

Maximum infusion requirement of phenylephrine and norepinephrine were 3.28 ± 1.02 μg/kg/min and 2.96 ± 0.28 μg/kg/min, respectively. There was significant increase in post-treatment levels of SBP, MAP, SVRI, VO_2_I, DO_2_I, and HVOS in both the groups [Table 4]. PAOP increased only in group 1. There was statistically significant decrease in post-treatment serum lactate in both groups (*P* < 0.05). There was statistically significant post-treatment reduction in HR (*P* < 0.001) and increase in SVI (*P* < 0.001) in group 1 [Table 4]. No significant change occurred in post-treatment levels of HR and SVI in group 2. There was no significant change in post-treatment CI in both groups. There was significant increase in post-treatment UO in both groups (*P* < 0.001) [Table 4]. The number of responders and survivors are described in Figure 1.

## Discussion

A distinctive characteristic of sepsis is intravascular volume depletion, decreased peripheral resistance, hypotension, and non-uniform distribution of regional blood flow. Aggressive volume resuscitation has been regarded as the first-line treatment in the management of sepsis.[4] However in the presence of substantial peripheral vasodilatation, patients may remain hypotensive regardless of adequate fluid resuscitation.[7] Vasoactive agents are needed in such patients, to restore systemic vascular tone and to ensure adequate tissue perfusion.[7] No consensus exists till now regarding the vasoactive agent of choice in patients with septic shock.

Dopamine has been considered as the first-line vasoactive agent, in the management of septic shock.[4,8] However there are concerns regarding tachyarrhythmia, elevated myocardial oxygen requirements, associated gut ischemia, and undesirable endocrine effects with the use of dopamine.[8] Previous studies, however, recommend the use of other presser agents only in patients who are “dopamine-resistant.”[4,9] Dopamine-resistant sepsis patients were included in our study taking this into consideration.

In the present study, no difference was observed between phenylephrine and norepinephrine, in terms of improvement in post-treatment hemodynamic parameters like SBP/MAP/SVRI. It may be due to α_1_ agonistic effect of both agents, leading to increase in SVRI and thereby SBP and MAP.[6,10,11] Norepinephrine is argued to be better than phenylephrine in terms of improvement in myocardial contractility, thereby CI, due to additional action on β_1_-receptors in volume-resuscitated patients.[12] However, additional β_1_-receptor stimulation in the presence of ongoing dopamine infusion will maintain a high HR and consecutive increase in CI will not be achieved, in absence of adequate cardiac filling. Phenylephrine on the other hand increases SVI at the expense of decrease in HR, with no consecutive improvement in CI as depicted by the results of this study.[3,11]

Baseline HR was higher in both groups; possible causes were persistent hypotension along with ongoing dopamine infusion. There were statistically significant improvements in HR (decrease) and SVI (increment), with phenylephrine in comparison to norepinephrine in our study. Patients on norepinephrine on the other hand demonstrated insignificant change in HR/SVI on account of additional β_1_ agonistic activity counteracting any decrease in HR by increased SVRI. So phenylephrine could be preferable over norepinephrine, since prolonged tachycardia may cause major cardiac events in critically ill patients.[13] Further it may have an additional advantage over norepinephrine in terms of improvement in myocardial oxygen supply–demand.[13]

Recent data suggest that tissue oxygenation is a major predictor of morbidity and mortality in patients of septic shock.[14,15] There was significant increase in post-treatment DO_2_I and VO_2_I parameters in both groups in the present study. It could be attributed to redistribution of blood flow to previously underperfused areas by both agents leading to better oxygen utilization.[3,9,11]

An increased serum lactate level is a usual feature of sepsis patients. It may be due to increased production or decrease in hepatosplanchnic circulation or both.[16] In the present study, there was significant decrease in serum lactate levels along with increased HVOS in both groups as compared to their pretreatment values. The justification could be the correction of splanchnic ischemia together with an efficient hepatic lactate uptake with the combined use of dopamine with test drug.[11,17] The addition of dopamine to norepinephrine, in patients on β-blocker therapy has been demonstrated in previous studies to increase intestinal perfusion.[18] The only previous study comparing phenylephrine and norepinephrine in sepsis did not showed improvements in blood lactate levels.[3] It could be due to nonusage of dopamine along with the test drug in the above study. Other studies show that norepinephrine infusion does not compromise splanchnic perfusion in the infusion range of 0.01–3.0 μg/kg/min in sepsis.[19,20] Studies with phenylephrine also show increased blood flow to the splanchnic circulation in sepsis patients in the dose range of 3.1 ± 1.0 μg/kg/min.[10,11,21] However, delayed administration of phenylephrine-replacing norepinephrine in septic shock patients has been argued in previous studies, to cause pronounced hepatosplanchnic vasoconstriction as compared with norepinephrine.[22,23] In our study, we did not observe such results, since phenylephrine infusion was started immediately after no response to adequate volume resuscitation and dopamine infusion (1 h). Maximal infusion rate of phenylephrine, required to achieve the target was 3.28 ± 1.02 μg/kg/min as compared to 2.96 ± 0.4 μg/kg/min of norepinephrine, respectively. Thus phenylephrine administration was comparable to norepinephrine, for the doses required to achieve targets in the present study.

There was significant increase in UO in both groups as compared to their pretreatment values. It may be due to increase in glomerular filtrate as a result of efferent arteriolar vasoconstriction with the use of both agents.[3] However, one must consider the fact that delayed administration of phenylephrine in sepsis may negatively affect renal function as compared to norepinephrine.[22]

Dopamine infusion was kept constant throughout the study duration (at a rate of 25 μg/kg/min), to prevent any bias in the results from any change in dopamine doses. Thus any decrease in dopamine infusion cannot be assessed as per protocol of this study. There were 20 responders in group 1 and 19 in group 2, respectively. Ultimately 12 subjects (out of 27) in group 1 and 11 in group 2 (out of 27) survived and were all responders. Thus these results indicate that no significant difference was observed in terms of patient outcome among both agents.

The limitation of present study is that inclusion criteria did not consider the requirement of other supportive measures like immune modulation, corticosteroids, renal replacement therapy into account. Another concern is the possibility of adverse metabolic alterations and decreased organ perfusion, with prolonged use of vasoconstrictor agents in septic shock; we are not able to delineate this aspect as our study duration was short. Clearly, additional clinical trials are needed to better clarify the role of vasopressor in the treatment of sepsis.

In conclusion, phenylephrine infusion is comparable to norepinephrine in reversing hemodynamic and metabolic abnormalities of sepsis patients, with an additional benefit of decrease in HR and improvement in SVI.
